# Pain That Challenges Survival: A Novel *SCN9A* Variant (p.Leu1623Gln) Causing Carbamazepine-Refractory Paroxysmal Extreme Pain Disorder in a Chinese Family — Case Report

**DOI:** 10.3390/reports9010017

**Published:** 2026-01-05

**Authors:** Man-Kwan Yip, Chun-Ying Janice Liu, Wing-Tat Poon

**Affiliations:** 1 Department of Clinical Pathology, Pamela Youde Nethersole Eastern Hospital, Chai Wan, Hong Kong, China; 2 Department of Anesthesiology, Perioperative and Pain Medicine, Pamela Youde Nethersole Eastern Hospital, Chai Wan, Hong Kong, China

**Keywords:** paroxysmal extreme pain disorder, *SCN9A*, Nav1.7, sodium channelopathy, carbamazepine-refractory, Chinese

## Abstract

**Background and Clinical Significance:** Paroxysmal extreme pain disorder (PEPD) is an extremely rare autosomal dominant sodium channelopathy caused by *SCN9A* gain-of-function variants. It is characterized by infantile-onset excruciating paroxysmal pain, typically in rectal, ocular, or mandibular regions, triggered by innocuous stimuli and accompanied by autonomic flares. Carbamazepine is dramatically effective in most reported cases. To date, only two genetically confirmed cases have been documented in Chinese patients, and fewer than 20 disease-causing variants are reported worldwide. We report the third Chinese case harboring a novel likely pathogenic *SCN9A* variant (p.Leu1623Gln), notable for its unusually severe, progressive, and carbamazepine-refractory phenotype, as well as life-threatening psychiatric sequelae, highlighting phenotypic heterogeneity and the devastating impact when standard therapy fails. **Case Presentation:** A Chinese male proband with positive family history presented with lifelong trigger-induced catastrophic burning and tearing pain in the perineum and lower limbs, associated with erythema, swelling, and occasional non-epileptic seizures. Attacks worsened with age despite escalating polypharmacy, including high-dose opioids, benzodiazepines, topical lidocaine and carbamazepine. Both the proband and his father developed profound psychosocial sequelae including severe depression and suicidal attempts. Next-generation sequencing in the proband revealed a novel heterozygous likely pathogenic variant NM_001365536.1 (*SCN9A*): c.4868T>A p.(Leu1623Gln). **Conclusions:** This third reported ethnic Chinese PEPD case expands the genotypic and phenotypic spectrum of *SCN9A*-related channelopathies, demonstrating that some variants can produce carbamazepine-refractory, progressive, and profoundly disabling disease with high suicidality risk. Early genetic diagnosis is critical in family planning and cascade testing, and has the potential in guiding targeted therapy that is under active research.

## 1. Introduction and Clinical Significance

Pain is normally a protective sensation, but mutations in the voltage-gated sodium channel (VGSC) Nav1.7 (encoded by *SCN9A*), which is a gatekeeper for pain, can transform it into a devastating lifelong disease. Gain-of-function mutations in *SCN9A* can produce two distinct inherited pain syndromes: primary erythromelalgia (PE) and paroxysmal extreme pain disorder (PEPD; OMIM #167400). PEPD, first described in the literature by Hayden et al. [1] in 1959 and also known as familial rectal pain syndrome, is characterized by infantile-onset attacks of excruciating burning pain, most commonly in the rectal, perineal, ocular, or mandibular regions, triggered by innocuous mechanical stimuli (e.g., defecation, flatus, cold, or touch), and accompanied by autonomic features such as skin flushing, lacrimation, and occasionally syncope or tonic–clonic movements [1,2]. Since the landmark description of 11 families and two sporadic cases with *SCN9A* variants by Fertleman et al in 2006 [3], only approximately 500 patients have been reported worldwide, predominantly in the UK and Netherlands [4]. However, clinically defined PEPD patients do not always harbor a *SCN9A* gene mutation [5]. The estimated incidence of PEPD is currently less than 1 in 1,000,000 cases, reflecting why fewer than 20 disease-causing variants are reported so far [6]. These variants typically cluster within the inactivation gate in loop 3 and the S4–S5 linkers in domains III and IV of Nav1.7, which serve as the receptor for the inactivation gate, causing impaired fast inactivation and persistent sodium currents [7].

As a sodium channelopathy caused by the hyperexcitability of nociceptive afferent fibers, carbamazepine, a non-selective sodium-channel blocker, is reported to dramatically reduce attack frequency and intensity in most of the published PEPD cases, making it the cornerstone of therapy [3,6]. However, carbamazepine-nonresponsive patients have rarely been documented [8,9,10,11], and the factors determining therapeutic response remain incompletely understood. To date, only two genetically confirmed PEPD cases of ethnic Chinese ancestry have appeared in the literature, raising the possibility of under-recognition in this locality [12,13].

Here we describe a Han Chinese family harboring a novel likely pathogenic *SCN9A* variant NM_001365536.1: c.4868T>A; p.(Leu1623Gln). This family represents the third genetically confirmed ethnic Chinese PEPD kin exhibit a carbamazepine-refractory, progressive phenotype with life-threatening psychiatric sequelae. This case report illustrated that (1) phenotypic heterogeneity in PEPD is greater than previously appreciated, notably in terms of clinical trajectory and therapeutic response to conventional treatment, (2) early genetic diagnosis is critical in avoiding potential opioid spirals and facilitating family planning and cascade testing, and (3) selective Nav1.7 antagonists currently in clinical development may offer hope for refractory cases.

## 2. Case Presentation

**Figure 1:**
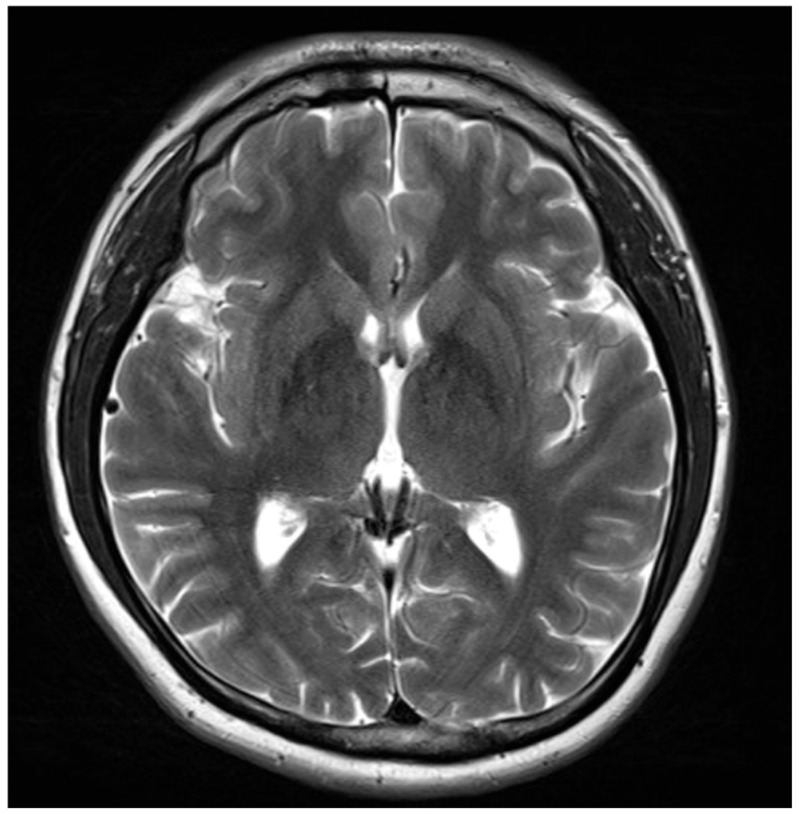
Magnetic resonance imaging of brain, which was normal.

A 30-year-old man had experienced extreme pain hypersensitivity below the T10 dermatome since birth. His first major attack occurred at age 15, triggered by passing flatus. Attacks consisted of sudden, tearing, crushing pain (with a numerical rating scale for pain of 10/10) affecting the perineum and bilateral lower limbs, lasting from minutes to hours, accompanied by visible erythema, swelling, tachycardia, and recurrent convulsions since age 26, with normal magnetic resonance imaging of brain at age 27 (Figure 1) and unremarkable electroencephalogram (the latest one performed at age 29). Minor facial attacks occurred more than 10 times daily with lacrimation, flushing, and rhinorrhea, each lasting for several minutes. Defecation and even anticipation of bowel movement reliably provoked attacks, resulting in chronic severe constipation. By 28 years of age, he reported up to three major flares daily requiring sublingual buprenorphine, with frequent emergency attendances for intravenous morphine. Pain progressively worsened, forcing him to quit his work at age 29. He was diagnosed by a psychiatrist to have major depressive disorder, and made several suicide attempts. Despite intensive polypharmacy from a multidisciplinary team consisting of pain specialists, neurologists, psychiatrists and clinical psychologists, including syrup morphine up to eight times daily, methadone, carbamazepine up to 600 mg daily (dosage limited by deranged liver function as well), mexiletine, pregabalin, multiple benzodiazepines, topiramate, fluoxetine, and topical lidocaine gel (up to five tubes daily), pain and mood control remained poor, complicated with opioid-induced hyperalgesia. Inhaled methoxyflurane, which is a general anesthesia not for unsupervised outpatient use, was once prescribed by a private pain specialist, but was subsequently stopped upon review of his condition. He is currently waiting for caudal epidural steroid injections and/or ganglion impar injections for advanced pain management.

Family history of extreme pain hypersensitivity and attacks was also reported in his paternal grandfather, father, and paternal aunt. His father had suffered identical symptoms since birth, with excruciating pain attacks associated with lower-body erythema, swelling, tachycardia, screaming, and double incontinence with preserved consciousness. For over 10 years he had tried oral midazolam, sublingual buprenorphine (later buccal fentanyl), methoxyflurane, zolpidem, and topical lidocaine or EMLA for the increasing severity and frequency of attacks, yet carbamazepine up to 800 mg daily was ineffective. He developed profound fear of pain attacks, social isolation, nightmares, and suicidal ideation since he was 65 years old. Regrettably, he committed suicide at the age of 70.

**Figure 2:**
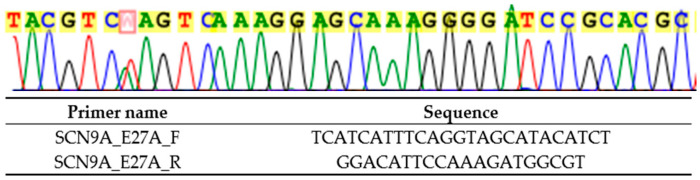
Sanger sequencing result (marked in red) and primers used (targeting *SCN9A* exon 27) for heterozygous NM_001365536.1 (*SCN9A*): c.4868T>A p.(Leu1623Gln).

Genetic testing performed in the proband in 2024 revealed a novel heterozygous variant NM_001365536.1 (*SCN9A*): c.4868T>A p.(Leu1623Gln), by next-generation sequencing with TruSight One clinical exome panel on NextSeq2000 (Illumina Inc., CA, USA), and confirmed on Sanger sequencing (Figure 2). This variant was classified as likely pathogenic by the American College of Medical Genetics and Genomics (ACMG) guidelines: PM1 (located in the highly conserved position in S4 of domain IV where PEPD-causing variants cluster), PM2_Supporting (absent from gnomAD v2.1.1 and v4.1.0), PM5 (different pathogenic missense substitution previously reported at the same residue, p.Leu1623Pro), PP3 (multiple in silico tools predict deleterious effect: REVEL 0.97, AlphaMissense 0.948), and PP4 (phenotype highly specific for *SCN9A*-related PEPD). The genetic finding is supportive of a genetic diagnosis of the exceedingly rare autosomal dominant *SCN9A*-related PEPD. Electrophysiological research has shown that the p.Leu1623Pro variant, affecting the same amino acid, depolarized the steady-state inactivation curve, increased ramp current significantly, and shortened recovery from inactivation, all of which are compatible with a gain of function in Nav1.7 [9]. In addition, amitriptyline, which is a non-selective sodium channel blocker, only slightly corrected the steady-state inactivation shift of the mutated channel [9], which may explain the lack of clinical benefit of carbamazepine in both this variant and our detected variant p.(Leu1623Gln). In addition, cooling was reported to be able to improve in the patient with p.Leu1623Pro, which is atypical in PEPD [9].

## 3. Discussion

**Figure 3:**
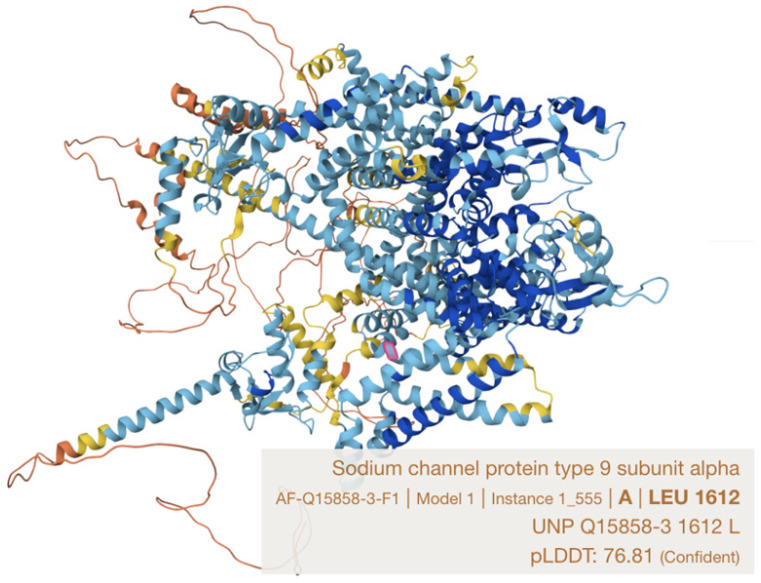
Leucine 1623 residue in Nav1.7 protein (Q15858-1), which is equivalent to leucine 1612 on Q15858-3 protein (marked in pink) (model constructed by AlphaFold).

Here we describe a single case report with family history in a Han Chinese family harboring a novel likely pathogenic *SCN9A* variant (NM_001365536.1: c.4868T>A p.(Leu1623Gln)). This variant locates in a recognized hotspot for PEPD-causing mutations, the S4 in domain IV, which is close to the cytoplasmic linker between S4–S5 in domains III and IV, being a docking site for the inactivation gate (with IFMT motif) of the sodium channel [9,14] (Figure 3). This family represents the third genetically confirmed ethnic Chinese (Table 1) and the first local kin group showing atypical severe features of PEPD in two affected family members with similar disease trajectory, characterized by carbamazepine refractoriness, requirement of intensive pain control, progressive worsening from childhood to late adulthood and potentially life-threatening psychiatric impact.

VGSCs play the primary role in generating electrical activity in nerve fibers by initiating and propagating action potentials. *SCN9A* encodes the VGSC Nav1.7, which is preferentially expressed in peripheral somatic and visceral sensory neurons, the nociceptive neurons at dorsal root ganglion, trigeminal ganglion, olfactory sensory neurons, and sympathetic ganglion [2]. Nav1.7 acts as a threshold channel that dramatically amplifies small depolarizations in nociceptors, making it essential for pain signal initiation and transmission [15]. Gain-of-function mutations cause two major autosomal dominant pain disorders distinguished primarily by their clinical symptoms: PE and PEPD [4]. In contrast, complete loss-of-function mutations abolish pain perception entirely, producing congenital insensitivity to pain (CIP) with anosmia (due to the expression of Nav1.7 in the olfactory epithelia) [2].

*SCN9A* gain-of-function mutations in PEPD can present in voltage-clamp recordings with (1) a hyperpolarizing shift in activation of the channel (making it easier to open the channel); (2) increased amplitude of ramp current (an enhanced response to slow, small depolarizations); (3) slower deactivation (slower closing of the channel when the stimulus is removed); and (4) depolarizing shifts in fast and slow inactivation (allowing more channels to be available to open) [5,16]. However, in vitro electrophysiological findings cannot reliably predict the clinical phenotype in a patient. The cellular context is critical, as illustrated by the fact that the same mutation can cause hyperexcitability in dorsal root ganglion neurons but hypoexcitability in sympathetic ganglion neurons. Furthermore, an individual’s genetic background and likely environmental factors also influences phenotypic expression, which explains why affected members of the same family can display diverse symptoms with varying onset and progression [5].

Unlike PE, pain in PEPD is a paroxysmal, mechanically triggered, autonomic storm centered on the perineum and lower body with typically excellent response to carbamazepine, whereas PE is a warmth-triggered, distal burning pain that is usually more constant or prolonged and less reliably controlled by sodium channel blockers. Both are *SCN9A* gain-of-function disorders, but the distinct biophysical effects of the mutations produce strikingly different clinical syndromes, though a patient complaining of symptoms characteristic of both diseases has been described (harboring the A1632E mutation) [5] (Table 2). PE has been more frequently reported in Chinese than PEPD, with 54 Chinese patients described so far, according to a recent review article [17].

Most PEPD patients respond dramatically to carbamazepine, which markedly reduces attack frequency and intensity [3]. Carbamazepine remains a first-line treatment for classic trigeminal neuralgia and is traditionally the drug of choice for PEPD. It is a state-dependent sodium channel blocker that preferentially binds to channels in their inactivated state and typically causes a negative shift in steady-state inactivation. However, this effect varies depending on the specific genotype and Nav channel subtype involved, some mutations (e.g., G1607A and L1612P) can be poorly responsive or completely resistant, while long-term use is frequently limited by side effects [5]. Therefore, we should avoid broadly stating that PEPD is carbamazepine-responsive, as this can create high expectations and patients may later be disappointed if the drug proves ineffective or causes intolerable side effects.

Other sodium channel blockers such as topiramate and lamotrigine can be helpful, while GABA-targeted drugs such as valproate or tiagabine offer only modest improvement. On the other hand, standard neuropathic pain medications remain reasonable options, including amitriptyline and gabapentin [5]. Opioid medications are typically not advised because they are often ineffective for pain control in PEPD, can worsen constipation [11], and have the potential to contribute to opioid spirals, leading to tolerance, dependence, and risk of overdose [18]. Newer sodium-channel blockers such as lacosamide and rufinamide show promise in preclinical studies and are worth trying [5]. Loss-of-function SCN9A mutations causing CIP genetically validate Nav1.7 as a high-priority analgesic target with minimal off-target effects. However, highly selective Nav1.7 inhibitors tested in humans fail to fully replicate CIP analgesia, with greater selectivity often yielding weaker pain relief. It is postulated that the pain insensitivity in CIP largely results from compensatory upregulation of endogenous opioids in sensory neurons upon complete Nav1.7 absence. It is thus proposed that future successful Nav1.7-based analgesics will likely need to target central terminals or combine Nav1.7 inhibition with modulation of the associated opioid signaling pathway [19,20].

To the best of our knowledge, only 17 PEPD disease-causing variants in *SCN9A* gene are described in the Human Gene Mutation Database (HGMD) 2025.03 (Phenotype ID: 336513054) (Table 3), most of them clustering in domains III–IV of Nav1.7 and impairing fast inactivation, producing persistent sodium currents that hyperexcite nociceptors. The extremely limited number of documented cases significantly impedes our ability to develop reliable and effective strategies for preventing these debilitating episodes of extreme pain. As a result, current management often remains reactive rather than proactive, with focus placed on treating attacks after they occur. This leaves patients vulnerable to unpredictable, severe suffering that can profoundly disrupt their lives, physically and mentally.

Switching to a new treatment requires time and carries the risk of side effects. Recent evidence shows that each unsuccessful treatment may reduce the likelihood of success with the next one [5]. Therefore, early genetic diagnosis (ideally with in vitro electrophysiological profiling or in silico modeling to guide treatment) and emerging selective Nav1.7 antagonists may offer hope for refractory cases and prevent inappropriate and futile opioid escalation [5]. We strongly advocate for expanded research efforts, including the creation of international patient registries and collaborative multicenter studies to better understand the underlying mechanisms of these rare but devastating pain crises. Only through such dedicated work can we hope to discover and refine truly effective preventive treatments and, ultimately, improve the quality of life for those suffering unendurable pain.

## 4. Conclusions

We report a Han Chinese family harboring a novel likely pathogenic *SCN9A* variant (NM_001365536.1: c.4868T>A; p.(Leu1623Gln)) that causes an exceptionally severe, progressive, and carbamazepine-refractory form of PEPD. This family, being the third genetically confirmed ethnic Chinese PEPD kindred reported, exhibited lifelong excruciating pain attacks with lower-limb and perineal predominance, and associated with non-epileptic tonic–clonic movements and life-threatening psychiatric comorbidity. In particular, the seven-decade ordeal of the proband’s father affirms that unrelenting extreme pain can indeed be fatal when hope is exhausted. This case report underscores three critical lessons: first, phenotypic heterogeneity in PEPD is far greater than previously recognized, with rare domain IV variants capable of producing devastating, treatment-resistant disease; second, early genetic diagnosis can be lifesaving, averting inappropriate opioid escalation, which is rarely effective in PEPD, enabling cascade testing and family planning, and preserving quality of life; and third, selective Nav1.7 antagonists currently in development may represent prospect of disease-modifying therapy for refractory patients. By integrating genomic insights with clinical phenotyping, precision approaches can potentially enable tailored strategies that address the underlying molecular pathology rather than relying solely on empirical symptom management for refractory PEPD, which is not merely disabling but can be lethal.

## Figures and Tables

**Table 1:** Three genetically confirmed PEPD cases in ethnic Chinese.

	Case 1	Case 2	Case 3
Origin	Guangdong	Zhejiang	Hong Kong
Year of reporting	2021	2022	2025
Sex	Male	Male	Male
Age of onset	5 months old	7 days old	Since birth
Age at report	7 months old	2 years and 6 months old	30 years old
Pain pattern	/	Paroxysmal attacks (minutes)	Paroxysmal attacks (minutes)
Main locations	/	Perineum and face	Perineum, bilateral lower limbs and face
Triggers	/	Defecation, touch, eating, and spontaneously during sleep	Defecation, flatus, touch
Autonomic signs	Apnea, bradycardia, skin color change, non-epileptic tonic seizures	Flushing, sweating, bradycardia, harlequin color change, non-epileptic tonic seizures	Flushing, swelling, tachycardia, lacrimation, non-epileptic tonic seizures
Carbamazepine response	Responsive	Responsive	Refractory
Variant detected (NM_001365536.1)	c.5273T>C; p.Val1758Ala	c.4417T>A; p.Phe1473Ile	c.4868T>A; p.Leu1623Gln
Remarks	De novo mutation absent in parents	Affected mother only showed bradycardia and sinus arrest	Increased severity and frequency of attacks with age
Reference	[12]	[13]	This case

**Table 2:** Key differences between PEPD and PE.

Feature	Paroxysmal Extreme Pain Disorder (PEPD)	Primary Erythromelalgia (PE)
*SCN9A* variant cluster	Mostly domains III–IV (impaired fast inactivation leading to persistent sodium current, with normal activation voltages)	Mostly domains I–II (hyperpolarizing shift in activation)
Age of onset	Infancy or early childhood	Childhood to early adulthood
Natural history	Usually stable or improves with age	Often worsens with age
Pain pattern	Paroxysmal attacks (minutes to hours)	Episodic or continuous burning pain
Main locations	Rectum/perineum, lower limbs, mandible, eyes	Distal extremities (feet > hands)
Triggers	Mechanical (defecation, flatus, touch, yawning, cold)	Warmth, exercise, standing, alcohol, spicy food
Autonomic signs	Intense flushing, swelling, tachycardia, lacrimation, harlequin changes, occasional tonic–clonic movements or incontinence	Warmth, redness, swelling of affected parts
Carbamazepine response	Dramatic reduction in frequency and intensity (rare refractory cases exist)	Variable; often partial or modest response
Other sodium channel blockers that may be effective	Mexiletine, topiramate, lacosamide	Mexiletine, lidocaine
Psychiatric comorbidity	High in severe/refractory cases	High in severe/refractory cases
Genetic diagnosis importance	Critical to avoid opioid escalation as rarely effective	Important, but cooling strategies remain mainstay

**Table 3:** Disease-causing variants described in HGMD 2025.03 for PEPD and their localizations.

	Disease-Causing Variants			Localizations (with Reference to Q15858-1, with 1988 Amino Acids)
	Transcript: NM_002977.3 (Used in HGMD)	Transcript: NM_001365536.1 (MANE Select)	Protein Changes	
1	c.554G>A	c.554G>A	p.Arg185His	S2–S3 linker of domain I
2	c.2986C>T	c.3019C>T	p.Arg1007Cys	Between S6 of domain II and S1 of domain III
3	c.3892G>T	c.3925G>T	p.Val1309Phe	S4–S5 linker of domain III
4	c.3893T>A	c.3926T>A	p.Val1309Asp	S4–S5 linker of domain III
5	c.3895G>T	c.3928G>T	p.Val1310Phe	S4–S5 linker of domain III
6	c.4382T>A	c.4415T>A	p.Ile1472Asn	IFMT motif between S6 of domain III and S1 of domain IV
7	c.4382T>C	c.4415T>C	p.Ile1472Thr	IFMT motif between S6 of domain III and S1 of domain IV
8	c.4384T>A	c.4417T>A	p.Phe1473Ile	IFMT motif between S6 of domain III and S1 of domain IV
9	c.4384T>G	c.4417T>G	p.Phe1473Val	IFMT motif between S6 of domain III and S1 of domain IV
10	c.4391C>T	c.4424C>T	p.Thr1475Ile	IFMT motif between S6 of domain III and S1 of domain IV
11	c.4819G>C	c.4852G>C	p.Gly1618Arg	S4 of domain IV
12	c.4835T>C	c.4868T>C	p.Leu1623Pro	S4 of domain IV
This case	c.4835T>C	c.4835T>C	p.Leu1623Gln	S4 of domain IV
13	c.4871T>C	c.4904T>C	p.Phe1635Ser	S4–S5 linker of domain IV
14	c.4880T>G	c.4913T>G	p.Met1638Arg	S4–S5 linker of domain IV
15	c.4880T>A	c.4913T>A	p.Met1638Lys	S4–S5 linker of domain IV
16	c.4895C>A	c.4928C>A	p.Ala1643Glu	S5 of domain IV
17	c.5218G>C	c.5251G>C	p.Val1751Leu	S6 of domain IV
18 #	c.5240T>C	c.5273T>C	p.Val1758Ala	S6 of domain IV

# The variant in Case 18 was not yet deposited in HGMD 2025.03 [12].

## Data Availability

The original contributions presented in this study are included in the article. Further inquiries can be directed to the corresponding author.

## Abbreviations

The following abbreviations are used in this manuscript:

VGSC	Voltage-gated sodium channel
PE	Primary erythromelalgia
PEPD	Paroxysmal extreme pain disorder
ACMG	American College of Medical Genetics and Genomics
CIP	Congenital insensitivity to pain
HGMD	Human Gene Mutation Database
